# Interactive Effects of Wide-Spectrum Monochromatic Lights on Phytochemical Production, Antioxidant and Biological Activities of *Solanum xanthocarpum* Callus Cultures

**DOI:** 10.3390/molecules25092201

**Published:** 2020-05-08

**Authors:** Hazrat Usman, Muhammad Asad Ullah, Hasnain Jan, Aisha Siddiquah, Samantha Drouet, Sumaira Anjum, Nathalie Giglioli-Guviarc’h, Christophe Hano, Bilal Haider Abbasi

**Affiliations:** 1 Department of Biotechnology, Quaid-i-Azam University, Islamabad 45320, Pakistan; usmanhazrat888@gmail.com (H.U.); asad_ullah8050@yahoo.com (M.A.U.); rhasnain849@gmail.com (H.J.); aisha_siddiquah@yahoo.com (A.S.); 2 Laboratoire de Biologie des Ligneux et des Grandes Cultures (LBLGC), INRA USC1328 Unversité ď, CEDEX 2, 45067 Orléans, France; samantha.drouet@univ-orleans.fr; 3 COSMACTIFS, Bioactifs et Cosmétiques, CNRS GDR3711, CEDEX 2, 4506 Orléans, France; 4 Department of Biotechnology, Kinnaird College for Women, Lahore 54000, Pakistan; sumaira.anjum@kinnaird.edu.pk; 5 EA2106 Biomolecules et Biotechnologies Vegetales, Universite de Tours, 37000 Tours, France; nathalie.guivarch@univ-tours.fr

**Keywords:** *Solanum xanthocarpum*, elicitation, multispectral lights, caffeic acid, coumarins, anti-diabetic, anti-inflammatory

## Abstract

*Solanum xanthocarpum* is considered an important traditional medicinal herb because of its unique antioxidant, and anti-diabetic, anti-aging, and anti-inflammatory potential. Because of the over exploitation linked to its medicinal properties as well as destruction of its natural habitat, *S. xanthocarpum* is now becoming endangered and its supply is limited. Plant in vitro culture and elicitation are attractive alternative strategies to produce biomass and stimulate biosynthesis of medicinally important phytochemicals. Here, we investigated the potential influence of seven different monochromatic light treatments on biomass and secondary metabolites accumulation in callus culture of *S. xanthocarpum* as well as associated biological activities of the corresponding extracts. Among different light treatments, highest biomass accumulation was observed in white light-treated callus culture. Optimum accumulation of total flavonoid contents (TFC) and total phenolic contents (TPC) were observed in callus culture kept under continuous white and blue light respectively than control. Quantification of phytochemicals through HPLC revealed that optimum production of caffeic acid (0.57 ± 0.06 mg/g DW), methyl-caffeate (17.19 mg/g ± 1.79 DW), scopoletin (2.28 ± 0.13 mg/g DW), and esculetin (0.68 ± 0.07 mg/g DW) was observed under blue light callus cultures. Compared to the classic photoperiod condition, caffeic acid, methyl-caffeate, scopoletin, and esculetin were accumulated 1.7, 2.5, 1.1, and 1.09-folds higher, respectively. Moreover, high in vitro cell free antioxidant, anti-diabetic, anti-aging, and anti-inflammatory activities were closely associated with the production of these secondary metabolites. These results clearly showed the interest to apply multispectral light as elicitor of in vitro callus cultures *S. xanthocarpum* to promote the production of important phytochemicals, and allow us to propose this system as an alternative for the collection of this endangered species from the wild.

## 1. Introduction

**Figure 1:**
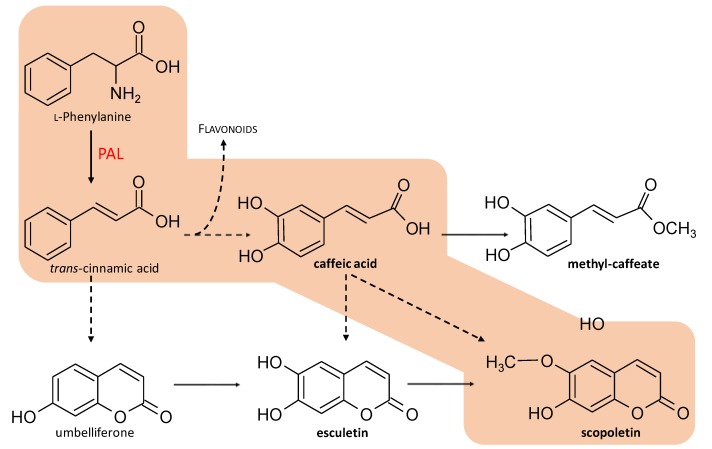
Schematic view of the phenylpropanoid pathway leading to the production of flavonoids, phenolics (in particular, caffeic acid and methyl-caffeate) and coumarins (in particular, esculetin and scopoletin). PAL: l-phenylalanine ammonia-lyse. Adapted from Bourgaud et al. [18] and Karamat et al. [19].

Medicinal plants are used by mankind as a source of primary health care for thousands of years, in an attempt to cure diseases because of their wide pharmaceutical and therapeutic applications [1]. Medicinal plants are used widely in both developed and developing countries. According to the WHO report on traditional medicine strategy 2014–2023, 70% population of the world rely on traditional medicines, mostly of plant origin, whereas 80% population of developing countries uses medicinal plants for treatment of diseases [1]. With a paradigm shift in the 20th century toward the use of synthetic chemicals, reputed to be more effective and safer to combat diseases, the interest in medicinal plants has temporarily failed and some studies reported that the use of medicinal plants was reduced by one-fourth during that period [2,3]. However, numerous studies show that the excessive use of synthetic drugs could have side effects and considerably increase the incidence of some diseases [3]. With the development of modern approaches, such as biotechnological plant sourcing, analytical tools for compounds identification, and isolation or molecular investigation of biological activity mechanisms, herbal medicine is back in the spotlights [4]. *Solanum xanthocarpum* Schrad. and Wendl (*Solanaceae*; aka *Solanum virginianum* L. or *Solanum mairei* H. Lév.) is a wild annual herb. The plant is also known under the vernacular names of Kantakari or Bhatkatiya. Its fruits, edible yellow berries surrounded by enlarged calyx, are largely used as traditional medicine to cure various ailments. Several biological activities relevant with their traditional uses have been reported, including antioxidant [5,6,7], anti-diabetic [8,9], anti-inflammatory [10,11], as well as effects on skin appearance [12]. Phytochemical profiling of *S. xanthocarpum* extracts revealed the presence of diverse classes of bioactive secondary metabolites including alkaloid glycosides [13,14], saponins [15,16], but also of high levels of coumarins [17], in particular of scopoletin and esculetin (Figure 1). From a biosynthetic point of view, these coumarins derived from the phenylpropanoid pathway, more precisely from *trans*-cinnamic acid, rather via umbelliferone than through lactonization reaction of the caffeic acid derivative, 2’-hydroxy-caffeic acid lead to esculetin formation [18]. Scopoletin is also produced from *trans*-cinnamic acid but via caffeic acid ester derivatives (Figure 1) [18,19]. Alternatively, caffeic acid is stored in its methylated form, i.e., methyl-caffeate, in *S. xanthocarpum* (Figure 1) [20].

Because of the over exploitation linked to its medicinal properties as well as destruction of its natural habitat, *S. xanthocarpum* is now becoming endangered and its supply is limited [21]. Therefore, more advanced research is needed to enhance both growth and secondary metabolites contents in this plant. Plant in vitro culture could represent an attractive option to multiply the plant [22], and also to produce plant biomass and secondary metabolites in controlled environment [23,24,25]. Light is an important abiotic elicitor that could also affect different physiological processes such as growth and development [26]. Therewith, changes in plant morphology and increased production of phytochemicals have been investigated previously in different species in response to multiple spectral lights applications in a controlled environment [27,28,29,30]. The current study was designed by emphasizing on establishing a new viable, fast, and effective protocol for optimum in vitro production of medicinally important phytochemicals from *S. xanthocarpum* in vitro cultures. For this purpose, several monochromatic spectral lights were investigated for their effects on both biomass and secondary metabolites productions. The cell-free in vitro antioxidant, antidiabetic, and anti-inflammatory potentials of each extract were also systematically evaluated to confirm the biological interest of the corresponding culture conditions.

## 2. Results and Discussion

### 2.1. Effects of Lights on Biomass Accumulation in S. xanthocarpum

In current study, leaf-derived callus of *S. xanthocarpum* was established on previously optimized phytohormonal balance (2.0 mg/L indole-3-acetic acid (IAA) + 0.5 mg/L 6-benzylaminopurine (BAP)) [31], and exposed to different monochromatic lights exposure.

**Figure 2:**
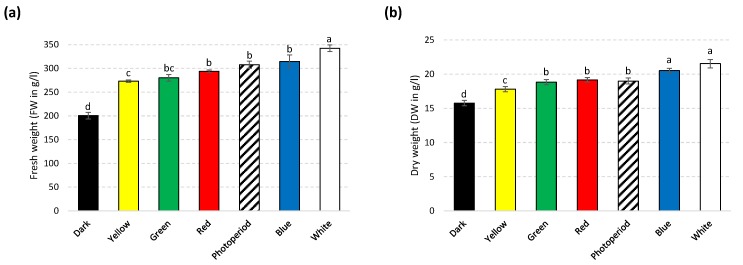
(**a**) Fresh weight (FW g/L) accumulation and (**b**) dry biomass (DW g/L) accumulation in callus cultures of *S. xanthocarpum* grown under different monochromatic lights conditions after 35 days of cultivation. Data are expressed as mean ± SD of at least three independent experiments. Different letters indicate significant differences between conditions (*p* < 0.05).

A significant variation was observed in biomass accumulation in tissues grown under different light sources. Maximum biomass accumulation was observed under white light (on both FW: 339.64 g/L and DW: 21.50 g/L basis) and blue light (on DW: 20.33 g/L basis) grown cultures as compared to photoperiod condition (FW: 304; DW: 18.81 g/L) (Figure 2a and b). However, significant inhibition in biomass accumulation was observed in cultures grown under dark (FW: 200.08, DW: 15.75 g/L), compared to rest of the light treatments.

**Figure 3:**
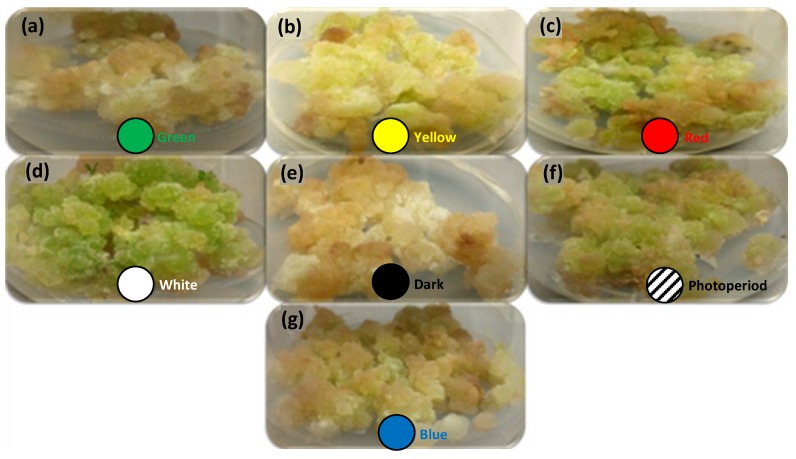
Representative effects of monochromatic lights on morphology of leaf explant-derived callus cultures of *S. xanthocarpum* grown under different monochromatic lights conditions after 35 days of cultivation.

Morphologically, all cultures were friable in nature and yellowish or light green in color (Figure 3). Greener callus color is a sign of higher chlorophyll production, leading to better photosynthetic potential. Overall, highest biomass production under white (FW and DW) and blue lights (DW) in callus culture of *S. xanthocarpum* was recorded. The effect of white light might be due to higher energy level resulting in higher photosynthetic rate [32], which is correlated with biomass production [33]. These findings are in agreement with previous reports of Tariq et al. [34], on *Artemisia absinthium* callus culture. Similarly, Ullah et al. [35] observed higher biomass accumulation in callus culture of *Lepidium sativum* under white light treatment. However, several reports are available on stimulation of biomass accumulation under white light in various medicinal plants [36,37,38,39,40,41]. Nonetheless, the addition of blue light (400–500 nm) not only stimulated biomass production in tomato (*Solanum lycopersicum*) but also enhanced photosynthetic capacity of tissues [42]. Although, red light (600–700 nm) is renowned due to efficient induction of photosynthesis, but growth and development of plants is seriously hampered (known as the “red light syndrome”) [42].

### 2.2. Total Phenolic and Flavonoid Contents in S. xanthocarpum

**Figure 4:**
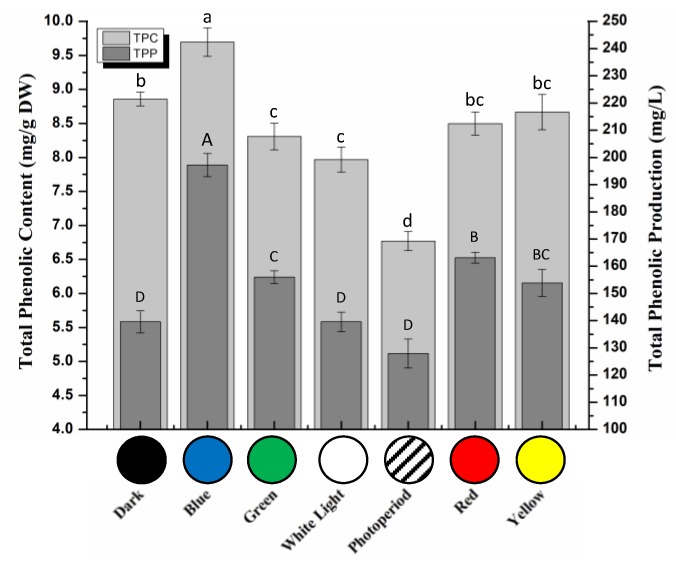
Total phenolic content (TPC) and total phenolic production (TPP) in callus cultures of *S. xanthocarpum* grown under different monochromatic lights conditions after 35 days of cultivation. Data are expressed as mean ± SD of at least three independent experiments. Different letters indicate significant differences between conditions (*p* < 0.05).

**Figure 5:**
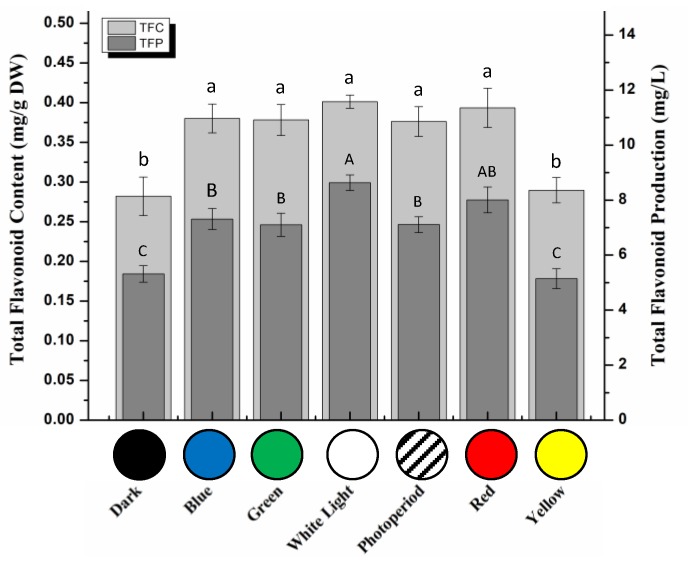
Total flavonoid content (TFC) and total flavonoid production (TFP) in callus cultures kept under different monochromatic lights. Data are expressed in terms of mean ± SD of at least three independent experiments. Different letters indicate significant differences between conditions (*p* < 0.05).

To cope with various environmental stresses, plant defense system is based on the production of low-molecular weight phytochemicals, including flavonoids and phenolics [43,44]. Phenolics and flavonoids are the key players to combat harsh conditions and regulate plant growth and development [45,46]. Total phenolics and flavonoids accumulations in callus culture of *S. xanthocarpum* under different lights treatment were evaluated (Figure 4 and Figure 5). Highest total phenolic contents (TPC: 9.69 mg/g) were observed for callus cultures grown under blue light condition (Figure 4). Total phenolic production (TPP) was also calculated based on respective dry weight production and TPC. Results indicated a similar trend i.e., maximum TPP was observed with blue light condition (197.18 mg/L). Several studies have highlighted the importance of blue light for enhanced production of secondary metabolites in various in vitro cultures from different species [37,47,48,49]. But, the effects of lights have been reported strictly depending on the plant species, type of culture, and nature of phytochemicals [35,36,39].

Contrarily, similar total flavonoid contents (TFC) were obtained in callus cultures grown under white (0.401 mg/g), red (0.393 mg/g), blue (0.380 mg/g), and green (0.378 mg/g) lights and photoperiod conditions (0.376 mg/g), whereas application of dark and yellow lights resulted in a significant down-regulated flavonoids accumulation (TFC: 2.82 mg/g and 0.289 mg/g, respectively) (Figure 5). Optimum flavonoids production (TFP) was measured in callus cultures grown under white light (8.632 mg/L), whereas lowest TFP were observed for callus cultures grown under yellow light (5.144 mg/L) and dark (5.320 mg/L). Continuous white light has been reported previously for elevated flavonoids biosynthesis in different plant species [35,37]. Contrarily, Kapoor et al. [50] observed maximum TFC under blue light-treated callus culture of *Rhodiola imbricate.*

In plant in vitro systems, light is used as an important abiotic elicitation strategy to enhance the biosynthesis of phytochemicals [51]. Callus cultures of several medicinal plant species have been exposed to monochromatic lights for enhanced production of secondary metabolites [35,37,51,52,53,54]. Here, the maximum accumulation of phenolics vs. flavonoids in different plants under different lights is in good agreement with the observation that the effect of the quality and intensity of light varies from one plant species to another. Light quality and intensity directly affect the enzymatic activity level of key enzymes of the different branches of the phenylpropanoid pathway. This could affect for example the activity of the PAL enzyme, the entry branchpoint enzyme between primary (l-Phe) and secondary (phenylpropanoid) metabolism (Figure 1), thus controlling the flux production toward the different classes of phenylpropanoid-derived compounds. But light quality and intensity could also differentially affect different internal branchpoint enzymes of the phenylpropanoid pathway (e.g., leading the accumulation of phenolics, coumarins, or flavonoids). To illustrate this point, recent gene expression and metabolic analyses have revealed distinct regulatory mechanisms involved in flavonoid and phenylpropanoid biosynthetic pathways in lettuce grown under different light quality and intensity conditions [55].

### 2.3. HPLC Analysis of Phytochemicals

**Figure 6:**
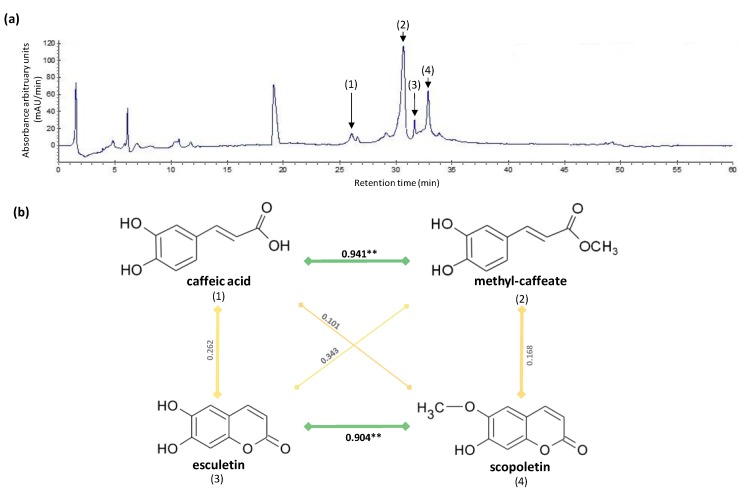
(**a**) Typical HPLC chromatogram showing the presence of caffeic acid (1), methyl-caffeate (2), esculetin (3), and scopoletin (4) in in vitro culture of *S. xanthocarpum.* (**b**) Correlation analysis (Pearson correlation coefficient, PCC) of the relation between caffeic acid, methyl-caffeate, scopoletin, and esculetin in in vitro culture of *S. xanthocarpum* subjected to different light treatments. Significance level: * *p* < 0.05, ** *p* < 0.01, *** *p* < 0.001.

In the current study, four compounds including two caffeic acid derivatives (caffeic acid itself and methyl-caffeate) and two coumarin derivatives (scopoletin and esculetin) were identified and quantified by HPLC in callus cultures of *S. xanthocapum* (Figure 6a). Data are presented in Table 1.

Highest amount of methyl-caffeate (17.19 ± 1.79 mg/g DW) was observed in blue light grown callus. Production of methyl-caffeate *ca* is 2-folds higher under blue light than photoperiod (Table 1). In case of quantitative analysis of caffeic acid biosynthesis, maximum production (0.57 ± 0.06 mg/g DW) was also observed in blue light-treated cultures. As mentioned, in plants, caffeic acid and their derivatives are synthesized through the phenylpropanoid pathway, which is activated by PAL enzyme [56,57]. According to Abbasi et al. [57], light treatment could enhance PAL activity and stimulate the biosynthesis of caffeic acid and its derivatives in hairy root culture of *Echinacea purpurea*. Nadeem et al. [37] as well as Khan et al. [39] reported optimum caffeic acid accumulation in *Ocimum basilicum* under red light and in *Fagonia indica* under white light, respectively. Iwai et al. [58] reported that exposure to blue light in combination with red light and UV rays have resulted in better accumulation of caffeic acid in *Perilla frutescens*.

The second class of phenylpropanoids quantified in current study is coumarins, which play a prominent role in plant defense against pathogens, oxidative stress, hormonal regulation, and response to different abiotic stresses [18]. Herein, two coumarins i.e., scopoletin and esculetin were quantified in callus cultures of *S. xanthocarpum* grown under different monochromatic light conditions. Optimum scopoletin accumulation (2.28 ± 0.13 mg/g DW) was observed in blue light-treated cultures as well as under red light and photoperiodic conditions (2.08 ± 0.09 and 2.07 ± 0.08 mg/g DW, respectively). Lowest scopoletin content was observed under yellow and white lights as well as dark conditions. Likewise, highest esculetin biosynthesis was noted in blue and red lights as well as photoperiod conditions. Similarly, Ogawa et al. [59] observed maximum coumarin accumulation in blue light-treated tea plant (*Camellia sinensis*). In contrast, highest accumulation of flavonolignans, another class of phenylpropanoids, in callus cultures of *Silybum marianum* was recorded under red light [60]. Taken together these observations confirm that, in response to different light quality and/or quantity, the complex phenylpropanoid pathway could simultaneously undergo differential activations vs. repressions of distinct branches leading to a differential accumulation of specific phenylpropanoids from one species to another. Here, significant correlations linking: (1) caffeic acid and methyl-caffeate (PCC = 0.941, *p* = 0.002), and (2) esculetin and scopoletion (PCC = 0.904, *p* = 0.005), were observed as a consequence of the different light treatments (Figure 6b) suggesting independent regulation of these two branches of the phenylpropanoid pathway in *S. xanthocarpum*.

### 2.4. In Vitro Antioxidant Activities

Exposure of plants to stress factors like pathogens, pollution, heavy metals, drought, salinity, extreme temperatures, high irradiance etc., causes a sudden shift in plant metabolic pathways resulting in higher production of ROS. ROS accumulation in higher amount could be harmful for plant cells and can damage biomolecules like proteins, carbohydrates, lipids, and DNA [61,62,63].

In response, plants produce oxidative enzymes and low molecular weight secondary metabolites, including various phenylpropanoids, to cope with the harmful effect of ROS leading to photo-oxidative damages [64,65,66,67].

In the literature, results showed significant increase in enzymatic antioxidant potential of calli extracts in response to light, which may result from PAL enzyme activation [68,69].

Antioxidant activity of plant extracts is based on complex mechanisms and is influenced/controlled by many factors that could influence extract composition. Therefore, it was deemed necessary to observe antioxidant potential of plant extracts by performing more than just one type of scavenging assay [66,70,71].

Notwithstanding, three distinct assays (i.e., 2,2-Diphenyl-1-picrylhydrazyl (DPPH), 2,2-Azinobis (3-ethylbenzthiazoline-6-sulphonic acid) (ABTS), and ferric-reducing antioxidant power (FRAP)) were performed to explore the antioxidant efficiency of *S. xanthocarpum* calli in response to different light regime. DPPH is a simple and widely accepted assay to evaluate the antioxidant activity of a compound or an extract [72].

**Figure 7:**
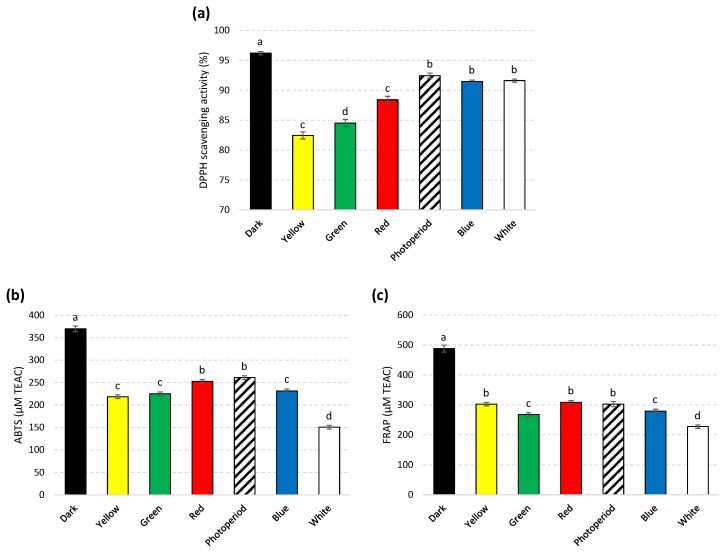
In vitro DPPH (**a**), ABTS (**b**), and FRAP (**c**) antioxidant activities of extracts from callus cultures of *S. xanthocarpum* grown under different monochromatic lights conditions after 35 days of cultivation. DPPH is expressed as % of radical scavenging activity (RSA). ABTS and FRAP are expressed in μM Trolox C equivalent antioxidant capacity (TEAC). The same volume of extraction solvent was used as blank. Data are expressed in terms of mean ± SD of at least three independent experiments. Different letters indicate significant differences between conditions (*p* < 0.05).

Results revealed that highest DPPH antioxidant potential (94.8% of radical scavenging activity (RSA)) was observed under dark conditions whereas yellow light exhibited lowest activity (81.0% RSA). Similarly, FRAP and ABTS activities were also found optimum in dark-grown cultures (514.88 μM and 356.17 μM Trolox C equivalent antioxidant capacity (TEAC) respectively) (Figure 7).

Standard antioxidant compounds, both synthetic antioxidants, like butylated hydroxyanisole (BHA) or butylated hydroxytoluene (BHT), or natural antioxidant, such as quercetin or secoisolariciresinol, have been reported to produce 90–99% RSA using DPPH [73,74].

Both FRAP (TEAC) and DPPH (% RSA) were reported for fruit extracts of *S. xanthocarpum* [5,7]. Our results obtained with FRAP (in TEAC) and DPPH (in % RSA) antioxidant assays of callus extract are in the range of the values reported for *S. xanthocarpum* fruit extract [5,7], thus evidencing the interest of our callus in vitro cultures as alternative to the wild grown plants of this endangered species [21]. Khan et al. [39] has previously concluded that callus culture is a rich source of antioxidant phytochemicals (phenolic and flavonoids). In this study, the authors observed blue light not only enhanced phytochemical production, but also had significant impact on the antioxidant activities of the calli extracts [39]. Similarly, higher antioxidant activities in response to blue light in callus culture of *Ocimum basilicum* and *Rhodiola imbricate* were previously reported [37,39]. Likewise, DPPH radical scavenging activity was observed maximum in in vitro cultured plantlets of *Rehmannia glutinosa* under blue light as compared to other spectrums by Manivannan et al. [75]. Contrarily, as in our case, higher antioxidant potential with light conditions, other than blue, has been reported for different plant cultures [38,48,51]. Once again, it is possible to directly link this observation to the effect of the quality and intensity of light that differentially impacts the production of various phytochemicals from one plant to another.

### 2.5. In Vitro Cell Free Anti-Diabetic Potential

**Figure 8:**
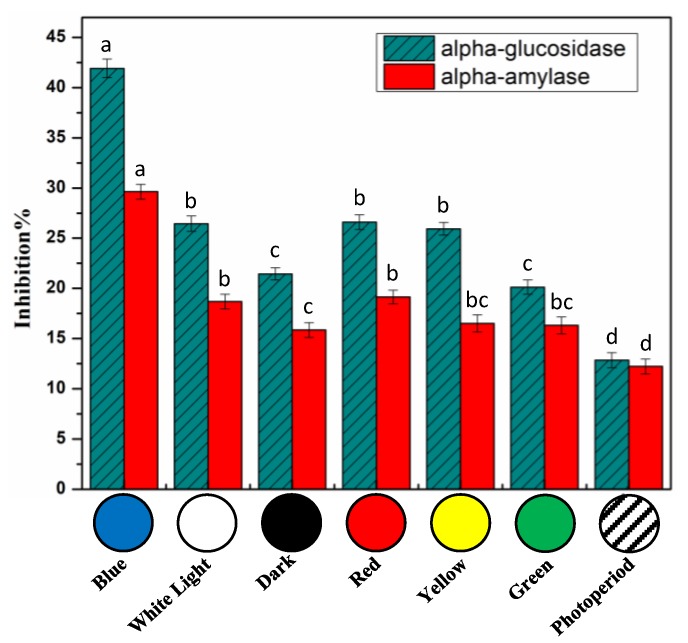
In vitro cell-free antidiabetic activity activities of extracts from callus cultures of *S. xanthocarpum* grown under different monochromatic lights conditions after 35 days of cultivation. Acarbose (10 µM) was used as positive control, leading to enzyme inhibition of 65.6 ± 4.1% and 89.2 ± 0.5% for α-glucosidase and α-amylase, respectively. The same volume of extraction solvent was used as blank. Data are expressed in terms of mean ± SD of at least three independent experiments. Different letters indicate significant differences between conditions (*p* < 0.05).

Diabetes is a chronic progressive condition caused by a deficiency in insulin secretion. Among the two types, type 2 diabetes is more prevalent. One of the treatments used for the remedy of type 2 diabetes is to control blood sugar levels after meal intake. This can be done through delaying glucose uptake by inhibiting enzymatic activity of intestinal α-glucosidase and/or pancreatic α-amylase [76,77]. In vitro cell-free α-amylase and α-glucosidase inhibition assays were performed to study the antidiabetic potential of *S. xanthocarpum* callus cultures (Figure 8). Light had a significant impact in enhancing the antidiabetic potential of calli extracts than photoperiod. Blue light had a greater efficiency against α-glucosidase (41.92%) and α-amylase (29.63%) inhibition compared to photoperiod (α-glucosidase: 12.86% inhibition and α-amylase: 12.23% inhibition) (Figure 8). For each experiment, acarbose (10 µM), the most commonly used therapeutic inhibitor, was used as positive control.

*S. xanthocarpum* fruits and leaves have been traditionally used for their potential anti-diabetic action, and anti-hyperglycemic activity of the corresponding extracts in animal models has reported [8,9]. Anti-hyperglycemic could be related to the inhibition of intestinal α-glucosidase and/or pancreatic α-amylase. The caffeic acid derivative, methyl-caffeate from *Solanum torvum* fruit extract showed significant reduction in blood glucose [20]. Caffeic acid and some of its derivatives showed marked inhibitory action on α-glucosidase [78] and α-amylase [79] as well as synergetic activity combined with acarbose [78]. Both esculetin and scopoletin displayed anti-hyperglycemic action in cellular and streptozotocin-(STZ-) induced diabetic animal models [80,81,82], and scopoletin showed a more pronounced inhibition activity against α-glucosidase and α-amylase than acarbose in STZ-induced diabetic mice [81].

Here, we show that in vitro culture extracts of *S. xanthocarpum* could exhibit potential anti-diabetic actions and be considered as an alternative to the wild grown *S. xanthocarpum*.

### 2.6. In Vitro Cell Free Anti-Aging Assays

Beneficial effects of *S. xanthocarpum* extract on skin appearance [12] in relation with its traditional use have been reported. In relation with the last two paragraphs, reactive oxygen species (ROS) were involved in the production of AGEs (advanced glycation end products), associated with cell aging and related disease especially in diabetic patients, but also skin appearance [83,84,85]. The anti-aging activities of plants have been attributed to their intrinsic capacity to reduce free radical damages to the skin, along with their aptitude to modulate the activity of various enzymes involved in aging process. Different secondary metabolites of plants are involved in inhibition of this process [35,86]. For instance, their capacity to inhibit elastase, hyaluronidase, collagenase, or tyrosinase. Collagenase, hyaluronidase, and elastase play a prominent role in the degradation of extracellular components of skin matrix, leading to the appearance of deep wrinkles and loss of resilience in the skin. On the other hand, any defect in tyrosinase can lead to pigmentary diseases such as melisma. Anti-aging potential of light-treated *S. xanthocarpum* callus extracts was, therefore, analyzed through their capacities to inhibit in vitro collagenase, hyaluronidase, tyrosinase, elastase, as well as AGEs (both vesperlysine-like and pentosidine-like AGEs) formations.

Compared to other light treatments, blue light appeared to be more practical for enhanced anti-AGEs formation activity of *S. xanthocarpum* callus extracts. Indeed, blue light treatment resulted in a significantly enhanced inhibition capacity of callus extract against the formation of both vesperlysine-like AGEs (45.36%) and pentosidine-like AGEs (47.08%). The results could have consequences on the anti-diabetic potential, but also the action on skin appearance, of the *S. xanthocarpum* callus extracts.

**Figure 9:**
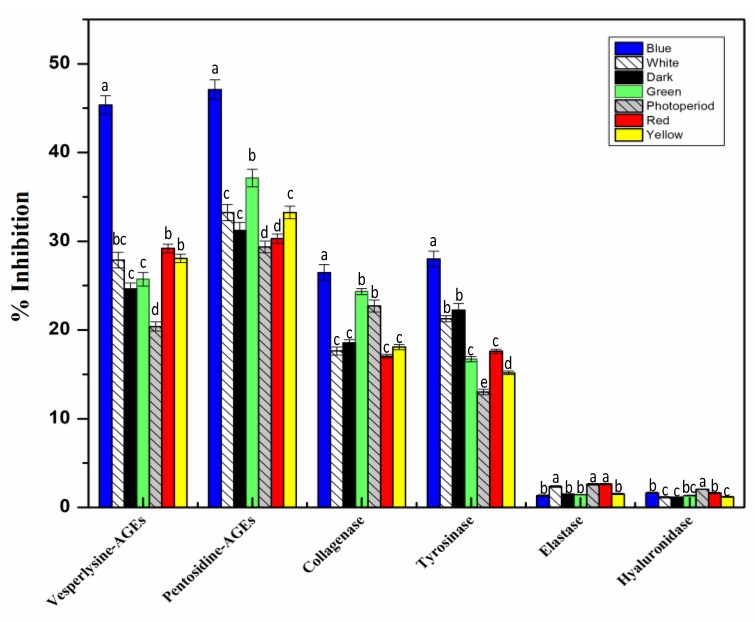
In vitro cell free anti-aging activity of extracts from callus cultures of *S. xanthocarpum* grown under different monochromatic lights conditions after 35 days of cultivation. Aminoguanidine (150 µM) was used as the standard inhibitor of AGEs formation leading to 27.3 ± 3.9% and 32.4 ± 4.2% inhibition of vesperlysine-like and pentosidine-like AGEs formation, respectively. 1,10-Phenantroline (100 µM) was used as the specific inhibitor of collagenase leading to an inhibition of 33.6 ± 2.2%. Kojic acid (10 µM) was used as the specific inhibitor of tyrosinase leading to an inhibition of 51.2 ± 0.9%. Oleanolic acid (10 µM) was used as the specific inhibitor of elastase and hyaluronidase leading to an inhibition of 47.8 ± 1.4% and 33.5 ± 2.8%, respectively. The same volume of extraction solvent was used as blank. Data are expressed in terms of mean ± SD of at least three independent experiments. Different letters indicate significant differences between conditions (*p* < 0.05).

Irrespective of the light treatment used, callus extracts of *S. xanthocarpum* did not present any effects on elastase and hyaluronidase enzyme activities (Figure 9). On the contrary, significant inhibitions of tyrosinase (27.99%) and collagenase (26.47%) activities were measured with extracts from callus grown under blue light (Figure 9).

To the best of our knowledge, this is the first study for analyzing anti-aging potential of in vitro derived callus cultures of *S. xanthocarpum* in response to different lights. Various studies are available in the literature documenting the anti-aging potential of elicited callus cultures of several plant species [35,86]. Caffeic acid and its derivatives have been reported as potent tyrosinase inhibitors [87], whereas esculetin and scopoletin showed significant potential toward the inhibition of collagenase [88,89].

### 2.7. In Vitro Cell Free Anti-Inflammatory Potential of S. xanthocarpum Callus Extracts

**Figure 10:**
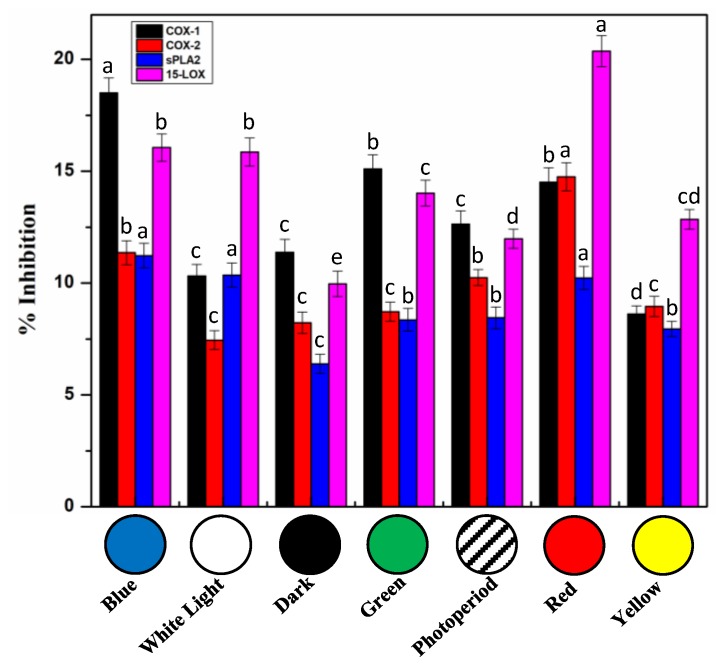
In vitro cell-free anti-inflammatory activity of extracts from callus cultures of *S. xanthocarpum* grown under different monochromatic lights conditions after 35 days of cultivation. Employed under the same experimental conditions, reference inhibitors were used as positive controls; Ibuprofen (10 µM) was used as positive control for COX-1 and COX-2 activity leading to enzyme inhibition of 31.4 ± 0.8% and 29.8 ± 1.2%, respectively; thioetheramide-PC (5 µM) was used as sPLA2 inhibitor, resulting in an inhibition of 43.7 ± 0.8%; nordihydroguaiaretic acid (100 µM) was used as 15-LOX inhibitor, leading to an inhibition of 30.6 ± 0.7%. The same volume of extraction solvent was used as blank. Data are expressed in terms of mean ± SD of at least three independent experiments. Different letters indicate significant differences between conditions (*p* < 0.05).

Inflammation is the local vascularized or immune response to harmful stimuli, pathogens, and irritants. Based on its traditional usages, anti-inflammatory action of *S. xanthocarpum* has been proposed [11,41]. Phenylpropanoids are one of the important classes of plants secondary metabolites that are involved in anti-inflammatory activities of plant extracts in relation with their capacity to inhibit key enzymes involved in inflammation process [38]. Anti-inflammatory action through a variety of mechanisms including, inhibition of cyclooxygenases (COX-1 and COX-2), phospholipase A2 (sPLA2), and lipoxygenase (15-LOX, eicosanoid generating enzymes), decreases leukotrienes and prostanoid concentrations [90]. Inhibitory potentials using in vitro cell free assays toward these four enzymes of *S. xanthocarpum* photo-treated callus extracts were evaluated (Figure 9). Moderate inhibition capacities were measured, but to be pointed, in comparison with values measured for the positive controls (Figure 10), and considering that crude extracts and not purified compounds were used. Extracts obtained from callus subjected to blue light treatment showed maximum inhibition activity toward COX-1 (18.51%), whereas extracts obtained from red light grown callus displayed the more pronounced inhibition activity of COX-2 (14.75%) and 15-LOX (20.37%). Phospholipase A2 (sPLA2) was less impacted, with maximum inhibition activity, around 12%, for extracts prepared from callus grown under red, blue, and white lights (Figure 10). Comparatively, the purified drugs, used as positive controls, Ibuprofen (10 µM) resulted in enzymatic activity inhibition of 31.4 ± 0.8% and 29.8 ± 1.2% of COX-1 and COX-2 respectively. Thioetheramide-PC (5 µM) resulted in a 43.7 ± 0.8% inhibition of sPLA2, and nordihydroguaiaretic acid (100 µM) led to an inhibition of 30.6 ± 0.7% of 15-LOX enzyme. On the contrary, extract derived from the callus grown under yellow light condition showed the overall lowest anti-inflammatory effect using these different in vitro cell-free assays (Figure 10).

These results are the first study documenting the effects of light on anti-inflammatory activity of extracts from *S. xanthocarpum* callus cultures. Although, anti-inflammatory activity form crude extract of wild plant has previously been studied in vivo and in vitro [10,11]. The effects of light on anti-inflammatory activity of extracts from in vitro cultures of *S. marianum* have been reported [38], thus confirming the feasibility of the production of anti-inflammatory compounds using plant in vitro biotechnological systems. From a mechanistic point of view, the potential anti-inflammatory actions of caffeic acid and its derivatives have been reported through the effective inhibition of both COXs [91] and 15-LOX [92] enzymes. Some coumarins showed inhibition against COXs activities, whereas scopoletin have been reported to significantly reduce inflammation [93] through the control of eicosnoid biosynthesis [94], and esculetin displayed a pronounced inhibition of 15-LOX [95].

### 2.8. Correlations Analysis

**Figure 11:**
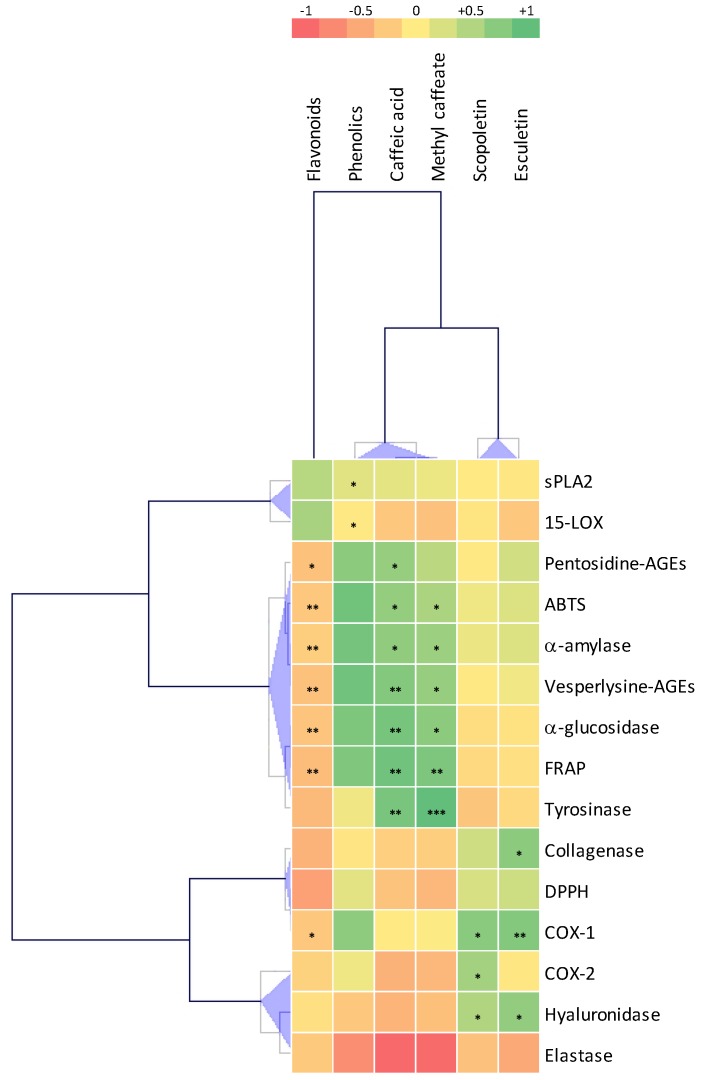
Correlation analysis (PCC) of the relation between the main phytochemicals (caffeic acid, methyl-caffeate, scopoletin, and esculetin) from extracts of in vitro culture of *S. xanthocarpum* subjected to different light treatments and the mentioned biological activities (antioxidant, anti-diabetes, and anti-inflammatory). Significance level: * *p* < 0.05, ** *p* < 0.01, *** *p* < 0.001. Actual PCC values are indicated in Supplementary Materials Table S1.

To better evaluate the connection between phytochemicals and biological activities of *S. xanthocarpum* extracts, Pearson coefficient correlations (PCCs) among these parameters were calculated (Figure 11; Table S1).

From this analysis, caffeic acid and methyl-caffeate appeared as the main contributor toward the FRAP (PCC = 0.829, *p* = 0.021 and PCC = 0.90, *p* = 0.007, respectively) and ABTS (PCC = 0.829, *p* = 0.021 and PCC = 0.770, *p* = 0.043, respectively) antioxidant activities, the potential anti-diabetic α-glucosidase (PCC = 0.909, *p* = 0.005 and PCC = 0.855, *p* = 0.014, respectively) and α-amylase (PCC = 0.840, *p* = 0.018 and PCC = 0.812, *p* = 0.027, respectively) enzymatic inhibitions, tyrosinase inhibitory action (PCC = 0.903, *p* = 0.005 and PCC = 0.963, *p* = 0.001, respectively), as well as inhibition of vesperlysine-like AGEs formation (PCC = 0.877, *p* = 0.010 and PCC = 0.826, *p* = 0.022, respectively). Caffeic acid was also significantly correlated with the inhibition of pentosidine-like AGEs formation (PCC = 0.825, *p* = 0.022). These results are in good agreement with literature data [5,7,20,78,79,87,96].

Both coumarins, here analyzed, i.e., esculetin and scopoletin, significantly correlated with COX-1 inhibition (PCC = 0.878, *p* = 0.009 and PCC = 0.859, *p* = 0.013, respectively) and hyaluronidase inhibition (PCC = 0.835, *p* = 0.019 and PCC = 0.758, *p* = 0.048), whereas only scopeletin was correlated with COX-2 inhibition (PCC = 0.790, *p* = 0.034) and only esculetin was correlated with collagenase inhibition (PCC = 0.854, *p* = 0.014). These observations are in line with literature data [88,89,93].

Both potentially anti-inflammatory sPLA2 and 15LOX inhibitions were correlated with TPC (PCC = 0.623, *p* = 0.048 and PCC = 0.520, *p* = 0.040), thus evidencing that further phenolics to be identified could be responsible for these inhibitions. Some phenolic compounds have been already reported for their capacities to inhibit both enzymes [97,98].

These results clearly show that in vitro culture extracts of *S. xanthocarpum* are not only considered as an alternative to the collection of *S. xanthocarpum* from the wild as a source of phytochemicals for traditional usages, but also represent an attractive model to study metabolic regulation of caffeic derivatives and coumarins.

## 3. Materials and Methods

### 3.1. Chemicals and Reagents

Exempt when mentioned, all chemicals and reagents were from Sigma-Aldrich (Sigma-Aldrich, Saint-Quentin Fallavier, France).

### 3.2. Seed Collection and Germination

Fresh seeds of mature *S. xanthocarpum* plant were collected from its wild habitat of district Malakand, Kp Province, Pakistan and were identified by plant taxonomist. A voucher specimen of the plant was deposited in the university herbarium (Quaid-i-Azam University, Islamabad, Pakistan). After collection, seeds were thoroughly washed with tap water to remove adhering dust particles. To determine the viability of seeds, water floating technique was employed. Viable seeds were separated and surface sterilized. For seeds sterilization, HgCl_2_ (0.1%) was used for 30 s and aqueous ethanol (70% (*v*/*v*)) for 1 min. Seeds were further rinsed three times with autoclaved distilled water in order to remove any adhering chemical which may cause problems in seed germination. Murashige and Skoog [99] (MS) media was used for inoculation by following Ahmad et al. [100] protocol. In brief, media was supplemented with agar (0.8% (*w*/*v*) as gelling agent) and 3% sucrose (as carbon source). After adjusting the pH at 5.6–5.7, media was autoclaved for 20 min at 121 °C temperature.

### 3.3. Callus Culture Establishment

*S. xanthocarpum* plantlets (30 days old) were used for the establishment of callus culture by using previously optimized protocol of Sheeba and Palanivel [31]. Briefly, leaf explants of 0.5 cm^2^ were inoculated on MS media fortified with PGRs i.e., combination of IAA and BAP (2 mg/L and 0.5 mg/L, respectively), for optimum growth and biomass accumulation. After inoculation, flasks were tightly plugged and kept in growth room at 25 ± 2 °C temperatures and photoperiod cycle of 16 h light and 8 h dark. After 21 days, callus was further sub-cultured on fresh media with respective hormonal concentration.

### 3.4. Lights Exposure Conditions

Seven monochrome lights of different wavelengths were used on *S. xanthocarpum* callus culture derived from leaf explant: blue light (460 nm), white light (400–700 nm), green light (510 nm), red light (660 nm), and yellow light (570 nm) applied in continuous. Photoperiod cycle using white light (400–700 nm) and dark (16 h light and 8 h dark). Continuous dark was also tested. Lights were retained at an intensity of 45–50 µmol/m^2^/s^1^, as measured by luxmeter in chamber (SU10, Jeiotech, Billerica, MA, USA). After 5 weeks, morphological differences were observed in calli. Harvested calli, from each treatment, were further used for the determination of biomass, biological assays, and phytochemical analysis.

### 3.5. Extracts Preparation

Modified protocol of Zahir et al. [101] was used to prepare callus extracts to evaluate flavonoid, phenolic contents and DPPH antioxidant activity. Harvested callus was dried for 24 h at 60 °C and ground to a powder by using mortar and pistol. Total of 100 mg of callus DW powder from each treatment was mixed thoroughly with 500 μL of methanol followed by vortexing for 10 min and sonication (ultrasonic bath USC1200TH, Prolabo, Sion, Switzerland) for 30 min. Whole process was repeated two times and then centrifuged for 10 min at 15,000 rpm. Supernatant was isolated for analysis of metabolite contents and biological assays.

### 3.6. Total Phenolic and Total Flavonoid Contents

To analyze the total phenolic contents of light-treated *S. xanthocarpum* callus extracts, slightly modified protocol of Singleton and Rossi [102], was followed by using Folin-Ciocalteu reagent. The reaction mixture was prepared by adding 20 µL of sample to 90 µL of both Folin-Ciocalteu reagent and sodium carbonate. After 5 min of incubation, OD reading was taken at 630 nm using a microplate reader (Multiskan GO, Thermo Fischer Scientific, Illkirch, France). Total phenolic content (TPC) was determined in mg/g DW gallic acid equivalent using a 5-points calibration curve (0–40 μg/mL; gallic acid; *R*^2^ = 0.998). Total phenolic production of all treated samples was calculated by using the following formula:

Total phenolic production (TPP, mg/L) = TPC (mg/g) × Dry Weight (g/L)(1)

Total flavonoid contents were determined through protocol of Ahmad et al. [100], with minor modifications by aluminum trichloride (AlCl_3_) colorimetric method. Total 200 µL of mixture was prepared in a microplate by adding 20 µL of methanol extracted sample followed by addition of 10 µL potassium acetate (98.15 g/L H_2_O), 10 µL AlCl_3_ (10% *w*/*v*), and 160 µL of distill water. Mixture was kept at room temperature (25 ± 2 °C) for 30 min absorbance was taken at 415 nm with micro plate reader (Multiskan GO, Thermo Fischer Scientific, Illkirch, France). Total flavonoid content was determined in mg/g DW quercetin equivalent using a 5-points calibration curve (0–40 μg/mL; quercetin; *R*^2^ = 0.998). Total flavonoid production was determined by the following formula:

Total flavonoid production (TFP, mg/L) = DW (g/L) × TFC (mg/g)(2)

### 3.7. HPLC Analysis

Detection and quantification of caffeic acid, methyl-caffeate, esculetin, and scopoletin produced in different callus cultures were performed through HPLC by using standard grade solvents. Varian HPLC system (Agilent Technology, Les Ullis, France) composed of Varian Prostar (230 pump; 410 autosampler; 335 Photodiode Array Detector) and a Metachem Degasit degasser controlled by Galaxie version 1.9.3.2 software. Separation of phytochemicals was done by following earlier documented method of Mozetič et al. [103]. The mobile phase was composed of solvent A = H_2_O with 0.2% (*v/v*) HCOOH (pH = 2.1) and solvent B = methanol used for separation through a 1-h run with the following gradient: 8% B (0 min), 12% B (11 min), 30% B (17–25 min), 33% B (28 min), 100% B (30−35 min), 8% B (36 min), and a flow rate of 1 mL/min. Hypersil PEP 300 C18 column (5 µm; 250 × 4.6 mm; Thermo Fischer Scientific, Illkirch, France) equipped with an Alltech guard column (10 × 4.1 mm; Thermo Fischer Scientific, Illkirch, France) was used with a separation temperature set at 35 °C. After each individual run, 10 min were used for re-equilibration. Identification and quantification were done using authentic standards of caffeic acid, esculetin, scopoletin (Sigma-Aldrich, Saint-Quentin Fallavier, France), and methyl-caffeate (LGC Standard, Molsheim, France) on the basis of retention time. Calibration curves (6 points, analytical range: 10–1000 µg/mL, 5 replicates) and analytical characteristics were: caffeic acid (y = 6.523x + 0.929, R^2^ = 0.9991), methyl-caffeate (y = 6.110x + 1.102, R^2^ = 0.9996), scopoletin (y = 7.403x + 0.683, R^2^ = 0.9991), and esculetin (y = 7.673x + 0.109, R^2^ = 0.9993), with(y) peak areas against (x) the injected amounts (in µg/mL). Quantities were expressed in mg/g DW. Examination for the considered samples was done in triplicates.

### 3.8. In vitro Cell-Free Antioxidant Assays

#### 3.8.1. DPPH Free Radical Scavenging Assay

DPPH reagent was used to determine the antioxidant activity of light-treated *S. xanthocarpum* callus extracts according to previously described protocol of Ahmad et al. [100]. Total of 200 µL of mixture was prepared by adding 20 µL of methanol extract to 180 µL of DPPH solution. After incubation of mixture in dark for approximately 60 min, absorbance was measured at a wavelength of 517 nm using microplate reader (Multiskan GO, Thermo Fischer Scientific, Illkirch, France). DPPH (180 µL) in combination with 20 µL DMSO and ascorbic acid with final concentrations at the rate of (40, 20, 10, and 05 μg/mL) were considered as negative and positive controls respectively. Activity was measured in terms of elimination of free radicals through the following formula

% scavenging DPPH radical activity (RSA) = 100 × (1−AE/AD)(3)

Here, AD is the absorbance of only DPPH solution without the addition of any sample, and AE represents the absorbance of samples with added reaction mixture at a wavelength of 517 nm.

#### 3.8.2. FRAP Assay (Ferric Reducing Antioxidant Power)

FRAP potential of light-treated cultures was determined via proposed protocol of Benzie and Strain [104], by using FRAP solution, composed of 300 mM acetate buffer, 20 mM of hydrated iron chloride (FeCl_3_.6H_2_O), and 10 mM of TPTZ. Callus extract (10 µL) was mixed with 190 µL of FRAP solution and kept for fifteen minutes at room temperature. Microplate reader was used to measure the mixture absorption at a wavelength of 630 nm (Multiskan GO, Thermo Fischer Scientific, Illkirch, France). Whole process was repeated thrice and antioxidant potential of samples was shown in terms of TEAC which stand for (Trolox C equivalent antioxidant capacity). The same volume of extraction solvent was used as blank.

#### 3.8.3. ABTS Assay

Protocol of Tagliazucchi et al. [105] with slight modifications was used to evaluate the ABTS potential of elicited cultures. Briefly, ABTS solution was prepared by mixing potassium persulphate (2.45 mM) to ABTS salt (7 mM) in equal proportion followed by 16 h incubation in dark. Absorbance of the solution was adjusted to 0.7 at a wavelength of 734 nm. Callus extract was thoroughly mixed with the solution and kept for 15 min at room temperature in dark. Absorbance of the reaction mixture was recorded using a microplate reader (Multiskan GO, Thermo Fischer Scientific, Illkirch, France) at 734 nm. Procedure of the activity was repeated three times and antioxidant activity was expressed in terms of TAEC. The same volume of extraction solvent was used as blank.

### 3.9. In Vitro Cell Free Anti-Diabetic Assays

#### 3.9.1. α-Amylase Inhibition Assay

A chromogenic method previously reported by Hano et al. [106], was followed to investigate α-amylase inhibition potential of calli extracts. The pancreatic α-amylase enzyme (1 U/mL; Sigma Aldrich, Saint-Quentin Fallavier, France) was prepared in (0.1 M, pH 6.8) phosphate buffer and thoroughly mixed with 4-nitrophenyl-α-d-maltopentaoside (5 mM) solution. An aliquot of the sample was then added to the reaction mixture and kept at 37 °C for half an hour. After incubation period, same volume of sodium carbonate (1 M) was added to terminate the reaction. The absorbance of the samples was then recorded using microplate reader at 405 nm (Multiskan GO, Thermo Fischer Scientific, Illkirch, France) and the activity was measured as % inhibition by finding the difference between absorbance in the presence and absence of the extract. Whole assay was repeated three times. Acarbose (10 µM) was used as the standard drug. The same volume of extraction solvent was used as blank.

#### 3.9.2. α-Glucosidase Inhibition Assay

To further assess the antidiabetic potential of calli extracts, intestinal α-glycosidase (Sigma Aldrich, Saint-Quentin Fallavier, France) inhibition assay was performed by a chromogenic method of Hano et al. [106] using polyethylene filter (0.45 μm) end-capped column. In short, an aliquot of test sample was added to the reaction mixture containing rat intestinal fluid (1 mL) consisting of (5 mM) 4NPG (4-nitrophenyl-α-d-glucopyranoside; Sigma) and incubated at 37 °C. After 30 min of incubation, (1 M) sodium carbonate solution was added to terminate the reaction. Absorbance of reaction mixture was recorded at 405 nm (Multiskan GO, Thermo Fischer Scientific, Illkirch, France) and activity was measured as % inhibition by finding the difference in absorbance value of both solutions i.e., in presence as well as in the absence of calli extracts. Whole assay was repeated three times. Acarbose (10 µM) was used as the standard drug. The same volume of extraction solvent was used as the blank.

### 3.10. In vitro Cell Free Anti-Aging Assays

#### 3.10.1. Inhibition of AGEs Formation

Inhibitory potential of all light-treated callus cultures against advanced glycation end products (AGEs) formation was determined according to modified protocol of Kaewseejan and Siriamornpun [107]. Sample extracts from each treatment were mixed with the solution of BSA (Sigma Aldrich) 20 mg/mL in the presence of phosphate (0.1 M) buffer at pH 7.4 comprising 0.02% NaN_3_ (sodium azide *w*/*v*). Prior to absorption reading at 330 nm (VersaFluor fluorometer; Bio-Rad, Marnes-la-Coquette, France), reaction mixture was placed in the dark for 5 days at 37 °C. The % inhibition was taken with respect to the corresponding control (adding the same volume of solvent) to each sample extracts. Aminoguanidine (150 µM) was used as the standard inhibitor of AGEs formation. The same volume of extraction solvent was used as blank.

#### 3.10.2. Elastase Inhibition Assay

Protocol of Wittenauer et al. [108] was adopted to determine elastase inhibition potential of calli extracts using enzyme porcine pancreatic elastase (Sigma Aldrich, Saint Quentin Fallavier, France) and N-Succ-Ala-Ala-Ala-p-nitroanilide as a substrate. Following release of *p*-nitroaniline activity was measured at 410 nm by using a microplate reader (BioTek ELX800; BioTek Instruments, Colmar, France). Elastase inhibition was measured in triplicates and expressed in % inhibition relative to control (adding same concentration of solvent mixture by replacing extract). Oleanolic acid (10 µM) was used as the specific inhibitor of elastase. The same volume of extraction solvent was used as blank.

#### 3.10.3. Tyrosinase Inhibition Assay

For analyzing tyrosinase inhibition potential of photo-treated cultures, slightly modified protocol of Chai et al. [109] was adopted by using L-DOPA as diphenolase substrate. In short, 5 mM L-DOPA was mixed with 50 mM Na_3_PO_4_ (sodium phosphate) buffer (pH 6.8) and 10 µL of *S. xanthocarpum* extract. At last, 0.2 mg/mL solution of mashroom tyrosinase (Sigma Aldrich, Saint Quentin Fallavier, France) was added to make the final volume of the mixture up to 200 µL. Whole mixture, without the addition of any treated extract, was used as control. Reading of the reacting mixture was taken at 475 nm by using microplate reader (BioTek ELX800; BioTek Instruments, Colmar, France). Tyrosinase inhibitory potential was indicated as % inhibition as compared to the corresponding control. Kojic acid (10 µM) was used as the specific inhibitor of tyrosinase. The same volume of extraction solvent was used as blank.

#### 3.10.4. Collagenase Inhibition Assay

Protocol of Wittenauer et al. [108] was adopted to determine collagenase inhibitory antiaging potential of calli extracts with the help of photo spectrometer by using enzyme Collagenase clostridium histolyticum (Sigma Aldrich, Saint Quentin Fallavier, France) and FALGPA (N-[3-(2-furyl) acryloyl]-Leu-Gly-Pro-Ala) as substrate. Decrease in FALGPA was observed for 20 min at 335 nm wavelength with the aid of microplate reader (BioTek ELX800; BioTek Instruments, Colmar, France). Data were measured in triplicates and anti-aging potential was expressed as % inhibition relative to control (adding same concentration of solvent mixture by replacing extract). 1,10-Phenantroline (100 µM) was used as the specific inhibitor of collagenase. The same volume of extraction solvent was used as blank.

#### 3.10.5. Hyaluronidase Assay

To determine the hyaluronidase inhibitory potential of light-treated extracts, protocol of Kolakul and Sripanidkulchai [110] was followed by using hyaluronidase (1.5 units, Sigma Aldrich, Saint Quentin Fallavier, France) enzyme and (0.03% (*w*/*v*)) hyaluronic acid solution as substrate. Hyaluronic acid was precipitated by (0.1% (*w*/*v*) BSA) acid albumin solution. Hyaluronidase inhibitory assay was measured with the aid of microplate reader (BioTek ELX800; BioTek Instruments, Colmar, France) at a wavelength of 600 nm. Inhibitory activity was measured as % inhibition according to the relative control (adding same concentration of solvent mixture by replacing extract). Oleanolic acid (10 µM) was used as specific inhibitor of hyaluronidase. The same volume of extraction solvent was used as blank.

### 3.11. Anti-Inflammatory Activities

COX1, COX2, 15-LOX, and sPLA2 inhibition assays were performed to study anti-inflammatory potential of *S. xanthocarpum* callus extracts treated with different monochromatic lights by employing protocol of Shah et al. [38].

#### 3.11.1. COX-1 and COX-2 Inhibition Assay

COX1 (ovine) and COX2 (human) assay kits were used according to the given instructions of manufacturer (701050; Cayman Chem; Co, Interchim, Montluçon; France). Arachidonic acid at concentration of 1.1 mM was used as substrate and 10 µM ibuprofen was used as the negative control. For measurement of activity, COX peroxidase component kit is used while Synergy II plate (BioTek Instruments, Colmar, France) was used to verify the oxidized C_10_H_16_N_2_ (tetramethyl-p-phenylenediamine, Wurster’s blue) at 590 nm using microplate reader (BioTek ELX800; BioTek Instruments, Colmar, France) for 5 min. Ibuprofen (10 µM) was used as the positive control for COX-1 and COX-2. The same volume of extraction solvent was used as blank.

#### 3.11.2. 15-LOX Inhibition Assay

The inhibitory activity of the samples against 15-LOX was also carried out by the kit method (760700, Cayman Chem. Co., Interchim, Montluçon, France) following the manufacturer’s instructions. The concentration of hydroperoxides produced during the lipo-oxygenation reaction was measured by the kit using standard filtered soybean 15-lipooxygenase in Tris-HCl buffer (10 mM) at pH 7.4. The absorbance was taken at 940 nm using microplate reader (BioTek ELX800; BioTek Instruments, Colmar, France). Thioetheramide-PC (5 µM) was used as sPLA2 inhibitor. The same volume of extraction solvent was used as blank.

#### 3.11.3. sPLA2 Inhibition Assay

Inhibition of phospholipase A2 (sPLA2) secretory was measured by kit method (10004883, Cayman Chem. Co, Interchim, Montluçon, France). Diheptanoyl thio-PC was used as a substrate, while thiotheramide-PC was treated as a positive control inhibitor. Absorbance was taken at 420 nm using microplate reader (BioTek ELX800; BioTek Instruments, Colmar, France). The % inhibition was examined by the given formula:

% Inhibition = [(IA − Inhibitor)/IA] × 100(4)

Here, inhibitor is labeled as activity of enzyme with inhibitor addition; IA as 100% activity of enzyme in the absence of inhibitor. Nordihydroguaiaretic acid (100 µM) was used as 15-LOX inhibitor. The same volume of extraction solvent was used as the blank.

### 3.12. Statistical Analysis

Whole experiment was carried out twice and in triplicates. Except when clearly mentioned in the figure legend, the significance at *p* < 0.05 means statistically significant and standard deviation was calculated by using Statistix 8.1 (Statistix, Tallahassee, FL, USA). All statistical analyses were performed with XL-STAT2019 (Addinsoft, Paris, France). Origin 8.5 software (OriginLab, Northampton, MA, USA) was used to generate graphics with their mean data values and standard errors.

## 4. Conclusions

Feasible platform for enhanced biosynthesis of secondary metabolites from *S. xanthocarpum* has been established. Physical elicitation with several monochromatic lights has shown considerable impact on biomass accumulation and secondary metabolites production. Furthermore, blue light has shown remarkable influence by stimulating the biosynthesis of total phenolic content, total flavonoid content, coumarins (esculetin and scopoletin), caffeic acid and its derivatives. Moreover, blue light enhanced in vitro cell-free antioxidant, anti-inflammatory, anti-diabetic, and anti-aging potential of *S. xanthocarpum*. These biological activities were closely associated with the production of secondary metabolites. Contrarily, continuous white light promoted biomass accumulation to a considerable extent. Hence, it is concluded that the exposure to multispectral light is a promising strategy to promote the production of important phytochemicals in *S. xanthocarpum*. These results clearly showed that in vitro culture extracts of *S. xanthocarpum* could be considered as an alternative to the collection of *S. xanthocarpum* from the wild as a source of phytochemicals for traditional use, and also represent an attractive model to study the metabolic regulation of caffeic derivatives and coumarins. However high throughput techniques like, whole genome sequencing can be applied for better understanding of the effect of monochromatic lights on these metabolic pathways of *S. xanthocarpum*.

## Figures and Tables

**Table 1:** Quantification of medicinally important phytochemicals in monochromatic light-treated callus cultures of *S. xanthocarpum.*

Light Treatment	Phytochemicals (mg/g DW)			
	Caffeic Acid	Methyl-Caffeate	Scopoletin	Esculetin
Photoperiod	0.32 ± 0.03 ^d^	6.64 ± 0.85 ^d^	2.07 ± 0.08 ^a,b^	0.62 ± 0.06 ^a,b^
Yellow	0.44 ± 0.01 ^c^	9.69 ± 1.33 ^c^	1.66 ± 0.06 ^c^	0.52 ± 0.05 ^b^
Red	0.40 ± 0.04 ^c,d^	9.73 ± 1.85 ^b,c,d^	2.08 ± 0.09 ^a,b^	0.56 ± 0.06 ^a,b^
Blue	0.57 ± 0.06 ^a^	17.19 ± 1.79 ^a^	2.28 ± 0.13 ^a^	0.68 ± 0.07 ^a^
White light	0.48 ± 0.01 ^b^	12.75 ± 1.41 ^b,c^	1.68 ± 0.07 ^c^	0.53 ± 0.04 ^b^
Dark	0.45 ± 0.04 ^b,c^	13.44 ± 1.75 ^a,b^	1.70 ± 0.11 ^c^	0.54 ± 0.05 ^b^
Green	0.43 ± 0.03 ^b,c^	9.33 ± 1.34 ^c^	1.88 ± 0.12 ^b,c^	0.56 ± 0.02 ^b^

Data are expressed in terms of mean ± SD of at least three independent experiments. Different letters indicate significant differences between conditions (*p* < 0.05).

## Supplementary Materials

The following are available online. Table S1: Actual values for PCC (Pearson Correlation Coefficient) showing the relation between the main phytochemicals (TPC, TFC, caffeic acid, methyl-caffeate, scopoletin and esculetin) from extracts of in vitro culture of *S. xanthocarpum* subjected to different light treatments and the mentioned biological activities (antioxidant, anti-diabetes, anti-aging and anti-inflammatory).

## Abbreviations

NAA	Naphthalene acetic acid
BAP	6-Benzyl adenine
DPPH	2,2-Diphenyl-1-picrylhydrazyl
HPLC	High-performance liquid chromatography
MS	Murashige and Skoog
TPC	Total phenolic content
TFC	Total flavonoid content
DW	Dry weight
FW	Fresh weight
PGRs	Plant growth regulators
ROS	Reactive oxygen species
PAL	Phenylalanine ammonia-lyase
FRAP	Ferric reducing antioxidant power
ABTS	2,2-Azinobis (3-ethylbenzthiazoline-6-sulphonic acid)
AGE	Advanced glycation end products
COX-1	cyclooxygenase-1
COX-2	cyclooxygenase-2
sPLA2	phospholipase A2
15-LOX	lipoxygenase
