# Supplementary material for: Interactive Effects of Wide-Spectrum Monochromatic Lights on Phytochemical Production, Antioxidant and Biological Activities of Solanum xanthocarpum Callus Cultures
Source: Molecules. 2020 May 8;25(9):2201. doi: 10.3390/molecules25092201 (PMC7248882; doi:10.3390/molecules25092201)
Supplement: Supplementary file 1 [file molecules-25-02201-s001.pdf]

Table S1. Actual values for PCC (Pearson Correlation Coefficient) showing the relation between the main phytochemicals (TPC, TFC, caffeic acid, methyl-caffeate, scopoletin and esculetin) from extracts of *in vitro* culture of *S. xanthocarpum* subjected to different light treatments and the mentioned biological activities (antioxidant, anti-diabetes, anti-aging and anti-inflammatory).

	TPC	TFC	caffeic acid	methyl-caffeate	scopoletin	esculetin
DPPH	0.620	-0.169	0.162	0.039	0.653	0.682
ABTS	0.925	0.208**	0.829*	0.770*	0.587	0.639
FRAP	0.885	0.096**	0.928**	0.890**	0.373	0.431
α-glucosidase	0.890	0.171**	0.909**	0.855*	0.411	0.462
α-amylase	0.917	0.265**	0.840*	0.812*	0.599	0.638
COX-1	0.845	0.195*	0.531	0.553	0.859*	0.878**
COX-2	0.586	0.303	-0.003	0.043	0.790*	0.503
sPLA2	0.623*	0.739	0.614	0.595	0.532	0.504
15LOX	0.520*	0.777	0.210	0.144	0.486	0.195
Verp. AGEs	0.927	0.150**	0.877**	0.826*	0.520	0.581
Pent. AGEs	0.853	0.152*	0.825*	0.728	0.520	0.667
collagenase	0.481	0.000	0.274	0.266	0.675	0.854*
tyrosinase	0.584	0.075	0.903**	0.963***	0.175	0.371
elastase	-0.355	0.215	-0.719	-0.698	0.143	-0.096
hyalunidase	0.204	0.445	0.009	0.133	0.758*	0.835*

TPC Total Phenolic Content; TFC Total Flavonoid Content; DPPH 2,2-Diphenyl-1-picrylhydrazyl radical scavenging activity ; ABTS 2,2-Azinobis (3-ethylbenzthiazoline-6-sulphonic acid) antioxidant activity; FRAP Ferric reducing antioxidant power; COX-1 cyclooxygenase-1 inhibition; COX-2 cyclooxygenase-2 inhibition; sPLA2 phospholipase A2 inhibition; 15-LOX lipoxygenase inhibition; Verp. AGE vesperlysine-like advanced glycation end products inhibition; Pent. AGE pentosidine-like advanced glycation end products inhibition.

Colors referred to the following PCC values:

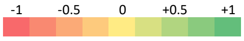

*p < 0.05, **p < 0.01, *** p < 0.001.
